# Unveiling Drought-Resilient Latin American Popcorn Lines through Agronomic and Physiological Evaluation

**DOI:** 10.3390/life14060743

**Published:** 2024-06-11

**Authors:** Uéliton Alves de Oliveira, Antônio Teixeira do Amaral Junior, Jhean Torres Leite, Samuel Henrique Kamphorst, Valter Jário de Lima, Rosimeire Barboza Bispo, Rodrigo Moreira Ribeiro, Flávia Nicácio Viana, Danielle Leal Lamego, Carolina Macedo Carvalho, Bruna Rohem Simão, Talles de Oliveira Santos, Gabriella Rodrigues Gonçalves, Eliemar Campostrini

**Affiliations:** Plant Breeding Laboratory, Center for Agricultural Science and Technologies (CCTA), State University of Norte Fluminense Darcy Ribeiro—UENF, Campos dos Goytacazes 28013-602, RJ, Brazil; uelitonalves2011@hotmail.com (U.A.d.O.); torresjhean@gmail.com (J.T.L.); valter_jario@hotmail.com (V.J.d.L.); rosimei-rebarboza1@hotmail.com (R.B.B.); rodrigo.moreira85@yahoo.com.br (R.M.R.); flaalegre@hotmail.com (F.N.V.); danieleallagemo@gmail.com (D.L.L.); carolinamacedocarvalho@gmail.com (C.M.C.); rohembruna@gmail.com (B.R.S.); tallesdeoliveira@live.com (T.d.O.S.); rdgabriella@gmail.com (G.R.G.); campostrini@uenf.br (E.C.)

**Keywords:** *Zea Mays* L. var. *everta*, leaf greenness, water deficit

## Abstract

Water stress can lead to physiological and morphological damage, affecting the growth and development of popcorn. The objective of this study was to identify the yield potential of 43 popcorn lines derived from a Latin American germplasm collection, based on agronomic and physiological traits, under full irrigation (WW) and water deficit conditions (WS), aiming to select superior germplasm. The evaluated agronomic traits included the ear length and diameter, number of grains per row (NGR) and rows per ear (NRE), grain yield (GY), popping expansion (EC), volume of expanded popcorn per hectare (VP), grain length (GL), width, and thickness. The physiological traits included the chlorophyll, anthocyanin, and flavonoid content in the leaves. The genetic variability and distinct behavior among the lines for all the agronomic traits under WW and WS conditions were observed. When comparing the water conditions, line L292 had the highest mean for the GY, and line L688 had the highest mean for the EC, highlighting them as the most drought-tolerant lines. A water deficit reduced the leaf greenness but increased the anthocyanin content as an adaptive response. The GY trait showed positive correlations with the VP, NGR, and GL under both water conditions, making the latter useful for indirect selection and thus of great interest for plant breeding targeting the simultaneous improvement of these traits.

## 1. Introduction

Global climate change is a major threat to life on Earth [1,2,3] due to the disruption of rainfall patterns and rising temperatures resulting from the increased greenhouse gas emissions [4,5,6,7,8,9]. In this scenario, drought is the main detrimental factor for agricultural production, with corn grain yield losses of 20 to 30% due to the water deficit [4,10,11]. Water scarcity is also a problem in many tropical and subtropical regions [4,12,13]. Thus, the lack of adequate rainfall and the increase in temperature limit the productive potential of crops, especially maize [4,14,15].

In cultivated crops, restrictions in the soil’s water availability trigger a cascade of changes at the morphological, physiological, and biochemical levels [16]. Morphologically, reduced turgor pressure leads to inhibited cell expansion, resulting in notable impacts on both shoot and root development, characterized by diminished height, decreased biomass synthesis, alterations in root structure, and premature leaf aging [17,18]. Leaves exhibit curling, reduced photosynthetically active surface area, and stomatal closure [19]. In an environment with soil drought, evaluating and monitoring the behavior of morphological and physiological traits can help in the selection of superior germplasm [16].

Agronomic traits and photosynthetic pigment indices are essential tools for evaluating maize genotypes under water deficit conditions [20,21,22,23]. Agronomic traits such as the grain yield and traits related to the ear and grain size allow the identification of plants that maintain good performance even with reduced water availability [24]. Meanwhile, photosynthetic pigment indices, such as the relative chlorophyll content, anthocyanins, and flavonoids, provide detailed insights into the photosynthetic efficiency and health of the plants [16,20,21,22,23]. Therefore, integrating these data offers a robust approach for selecting and developing maize genotypes that are more tolerant to water deficit.

Popcorn, a market that is worth more than USD one billion per year in the USA, is a crop of great economic interest that needs scientific advances aimed at developing cultivars adapted to soil water limitation. In Brazil, for the southeast region, only one hybrid with greater water use efficiency has been registered with the Ministry of Agriculture, Livestock and Supply [25], and there is a lack of other genotypes that can supply the national agribusiness. It is known that water deficit affects the growth and development of the crop and that water availability is crucial during flowering and grain filling of this species [26,27], with significant losses occurring when there is stress in these phases [4,10,11]. Developing popcorn genotypes that are more efficient in their use of water and identifying traits to help with selection under drought conditions are strategies that are urgently needed [28,29].

In popcorn, it is known that, besides agronomic traits, indices related to the leaf greenness, leaf photosynthetic status, and stomatal conductance have been shown to be reliable indicators of agronomic performance under drought [12,30,31,32,33,34,35,36]. In these studies, a set of Brazilian germplasms was used, making it necessary to expand the genetic base to be evaluated to identify new sources of superior germplasm. As a result, a collection of popcorn germplasms was set up with populations from different Latin American countries, from which S_7_ lines were obtained to investigate the responses of agronomic and physiological traits under contrasting conditions of water availability, i.e., water deficit (WS) and well-watered (WW) regimes, with the intent to select germplasms with an advanced inbreeding degree for the constitution of superior hybrid combinations. The novelty of this research lies in the fact that, for the first time, under drought conditions, the responses of agronomic and physiological traits have been evaluated in a sample of popcorn germplasms covering the whole Latin America, including different climatic adaptations (temperate and tropical), coming from both highlands and lowlands.

## 2. Materials and Methods

### 2.1. Genotypes

Forty-three popcorn lines from the Germplasm Bank of the Universidade Estadual do Norte Fluminense Darcy Ribeiro (UENF) derived from temperate and tropical genealogies were evaluated. They represent, above all, the genetic diversity of Latin America (Supplementary Table S1).

### 2.2. Experimental Conditions

The experiment took place during the 2019/2020 crop season in the experimental area of the Colégio Agrícola Antônio Sarlo (2°34′31″ S, 4°54′40″ W) in Campos dos Goytacazes, RJ, Brazil. The experiment was conducted under different water conditions: (i) well-watered (WW), with full irrigation recommended for the crop; and (ii) water deficit (WS), with irrigation suspended 15 days before male anthesis (CODE 61, state 6, BBCH Scale) until physiological maturity (CODE 99, state 9, BBCH Scale).

**Figure 1:**
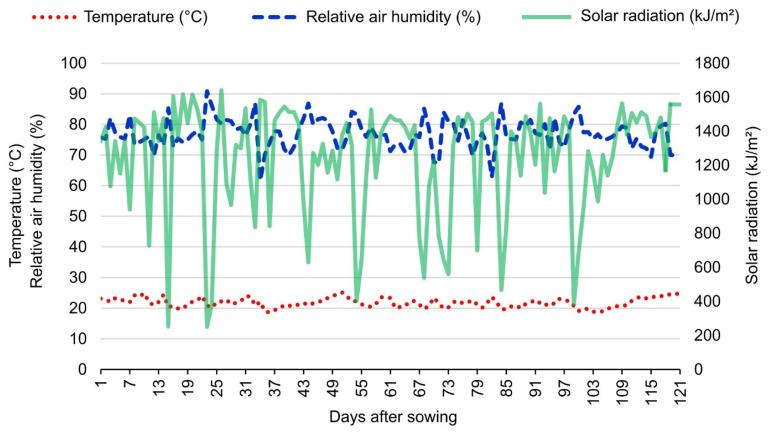
Climatic conditions of the temperature (°C) (red dotted line), relative air humidity (%) (blue dashed line), and solar radiation (kJ/m^2^) (green continuous line) recorded by the automatic station of the INMET from April to August 2020.

The weather conditions were monitored and quantified using an Automated Weather Station (A607) from the National Meteorological Institute (INMET) (Figure 1). During the experiment (April to August 2020), the average temperature (°C) per day ranged from 18.61 to 25.12, and the humidity from 61.61% to 90.84%. Solar radiation had a maximum of 921.06 and a minimum of 140.38 (µmol/m^2^s) (Figure 1).

The experimental design consisted of randomized blocks with three replications. Each experimental plot consisted of a row 4.40 m long, spaced 0.20 m between plants and 0.80 m between rows, totaling 23 plants per plot. A drip irrigation system was installed at the site, including one Katif dripper per plant with a flow rate of 2.30 mm h^−1^. This system enabled precise control of the amount of water applied to the plants, which was monitored using hydrometers installed in each water condition.

The lines were sown using 3 seeds per hole, with fertilization using NPK 04-14-08, corresponding to 30 kg ha^−1^ of N (in the form of urea), 60 kg ha^−1^ of P_2_O_5_ and 60 kg ha^−1^ of K_2_O. Thinning was conducted 28 days after emergence, leaving just 1 plant per hole, giving 23 plants per plot. At 30 days after sowing (CODE 13, state 1, BBCH Scale), topdressing was applied to the planting line with NPK 20-0-20, providing 60 kg ha^−1^ of N.

During the experiment, 137.84 mm of water was applied in the irrigated condition (WW), while 52.31 mm was supplied in the water deficit condition (WS), both added to the 117.0 mm of rainfall recorded during the experiment. The WS condition received 85.53 mm less water throughout the experiment than the WW condition (Table 1).

**Figure 2:**
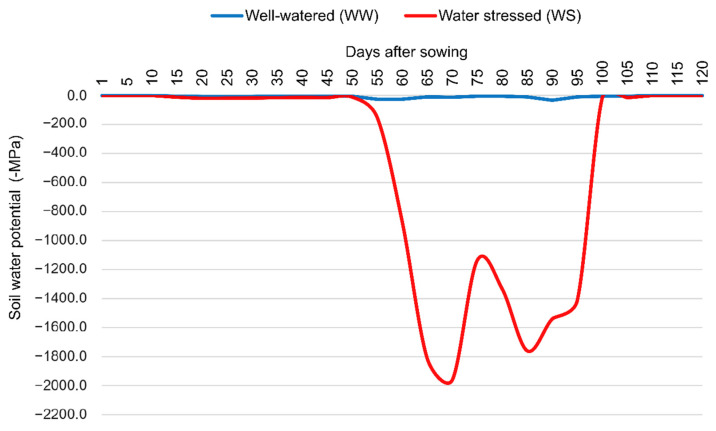
Soil water potential (WPa) in days after sowing (DAS) with popcorn lines under irrigated conditions (solid blue line) and water deficit (dashed red line). CODE 61, state 6, BBCH Scale. Equivalent to 60 days after sowing.

The soil water potential was monitored using five Decagon MPS-6 tensiometers (Decagon, Pullman, WA, USA), which were strategically distributed among the different WCs. The tensiometers were installed in the planting line between 2 plants at a depth of 0.20 m. In WS, where irrigation was suspended 15 days before male anthesis, the permanent wilting point (−1.5 MPa) was reached 63 days after sowing and 3 days after male flowering (CODE 61, state 6, BBCH Scale) (Figure 2).

### 2.3. Agronomic and Physiological Traits

The agronomic traits assessed were as follows. (i) Average ear length (AEL). (ii) Average ear diameter (AED). (iii) Number of rows of grains per ear (NRE). (iv) Number of grains per row (NGR). (v) Grain yield (GY), where the GY yield was estimated in grams per plot, corrected to 13% humidity and expressed in kg.ha^−1^. (vi) The 100-grain weight (100 W), where 100 grains of each genotype were counted with 3 repetitions and then quantified on an analytical balance with a resolution of 0.001 g. (vii) Expansion capacity (EC), which was measured for the mass of 30 g of grains, irradiated in a microwave, in a special paper bag for popping, at a power of 1000 W, for a set time of one minute and forty-five seconds. The volume of popcorn was quantified in a measuring cylinder (mL). The EC was determined by the quotient of the volume of popcorn obtained and the mass of the grains, expressed in mL g^−1^. (viii) The volume of expanded popcorn per hectare (VP) was obtained by multiplying the GY and EC, expressed per hectare m^3^ ha^−1^.

A grain trait was the caryopsis circularity index (CCI), which was estimated using the equation CCI = GT/GW + GL, where “GT” was the average grain thickness, “GW” was the average grain width, and “GL” the average grain length. To accomplish this, the length, thickness, and width of 15 grains were measured, with 3 repetitions per sample, using a digital caliper. The thickness/width ratio (TWR) was determined by the ratio between the average length of the grain and the average width of the grains, based on the equation TWR = E/L. For this purpose, a sample of 15 grains was used for each genotype, with 3 repetitions.

**Figure 3:**
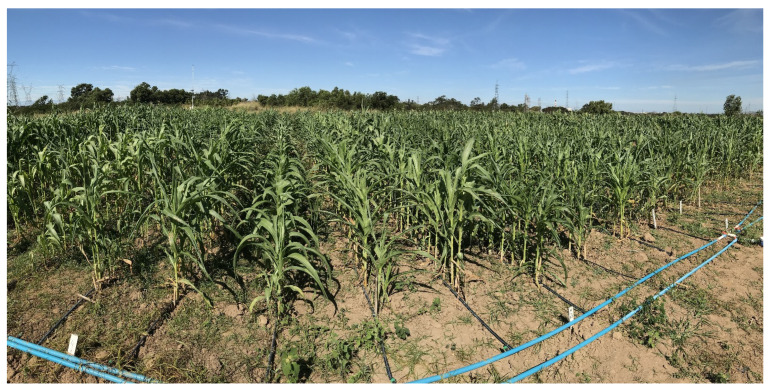
Panoramic image of the experiment under the water deficit condition ten days before male anthesis.

The physiological traits assessed were the relative chlorophyll content (SPAD), measured using a SPAD-502 portable chlorophyll meter, leaf anthocyanin content (ANT) and flavonoid content (FLV), which were measured using a Scientific Dualex^®^ ((FORCE-A, Orsay, France))portable chlorophyll meter (model FORCE-A). The average for each plot was calculated from 10 measurements, which were automatically generated by the equipment. The evaluations were conducted ten days before male anthesis (−10) (Figure 3), during the anthesis period (0 = CODE 61, state 6, BBCH Scale), and three evaluations after the anthesis period [(10), (20), and (30)], at 10, 20, and 30 days, respectively.

### 2.4. Statistical Analysis

For the agronomic traits, an individual analysis of variance was carried out for each water condition—WW and WS—based on the following statistical model: $Y_{ij}=\mu +G_{i}+B_{j}+\varepsilon_{ij}$, where $Y_{ij}$ corresponds to the observation of the i-th genotype in the j-th block; μ is the general constant; $G_{i}$ is the fixed effect attributed to the i-th genotype; $B_{j}$ is the random effect of the block; and $\varepsilon_{ij}$ is the random error associated with observation $Y_{ij}$, being NID (0, σ²). The a joint analysis was carried out by considering the following linear model: $Y_{ij}=\mu +G_{i}+{WC}_{j}+{GWC}_{ij}+{B/WC}_{jk}+\varepsilon_{ijk}$, in which the mean (µ), the genotype effect (G_i_), and the water condition effect (WC_j_) were considered to be fixed, and the block within condition effect (B/W_jk_) and the error (ε_ijk_), as random

Subsequently, only for the agronomic variables, the genotypic correlation coefficient (rg) estimates were estimated according to Mode and Robinson (1959) [37] and then tested for the 5% and 1% probability levels using the *t*-test. Regression analysis was conducted for the physiological traits, in which the lines were classified as superior (average of ten lines with the highest values), all (average of all the lines), and inferior (average of ten lines with the lowest values) based on the GY trait. Subsequently, the group means of the physiological traits were subjected to quadratic regression analysis, which was conducted using the RStudio software V4 [38] using the *ggplot2* package [39].

## 3. Results

### 3.1. Analysis of Variance, Mean Estimates, and Impact of Water Limitation on Morpho Agronomic Traits

The individual analysis of variance revealed significant differences (*p* < 0.01) for all the traits evaluated under both water conditions (WCs) (Table 2). In the joint analysis, the CCI and TWR traits showed significance at *p* < 0.05, with no significance for the GT. The other traits were significant at *p* < 0.01. All the traits showed significant G * WC interactions at *p* < 0.01, except for 100 W, which showed significance at *p* < 0.05 (Table 2).

The experimental coefficient of variation (CVe) showed percentages below 20% for all the traits in WW; in WS, it showed values above 20% for the GY and VP traits.

**Figure 4:**
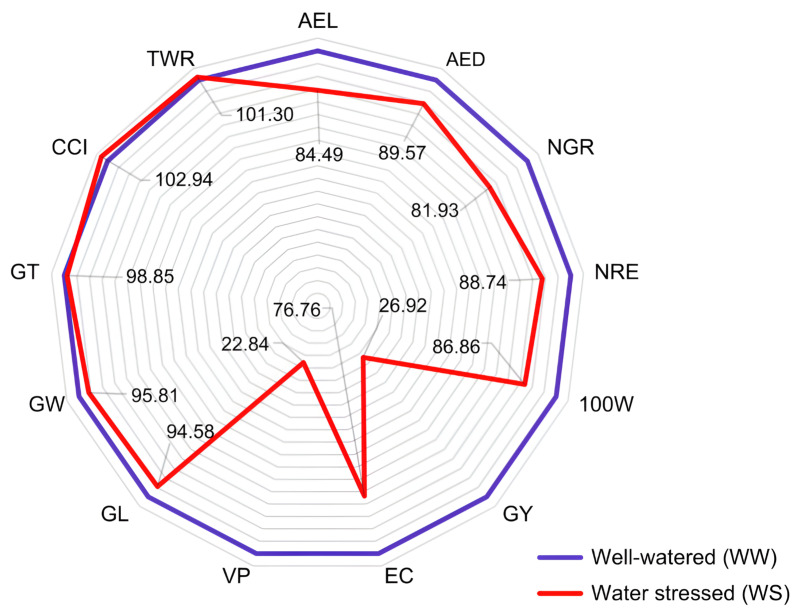
Relative percentages of the averages obtained under well-watered conditions (WW) compared to those obtained under water deficit conditions (WS) for 13 agronomic traits evaluated in 43 popcorn lines. AEL: average ear length; AED: average ear diameter; NGR: number of grains per row; NRE: number of grain rows per ear; 100 W: 100-grain weight; GY: grain yield; EC: expansion capacity; VP: volume of expanded popcorn per hectare; GL: grain length; GW: grain width; GT: grain thickness (mm); CCI: caryopsis circularity index; TWR: thickness/width ratio.

The agronomic traits AEL (−15.51%), AED (−10.43%), NGR (18.07%), NRE (11.26%), 100 W (−13.14%), GY (−73.08%), EC (−23.24%) and VP (77.16%) were those most affected by drought, with magnitudes above 10% (Figure 4). The VP, GY, and EC traits were the most affected under water deficit in absolute values. The VP showed an average of 33.58 m^3^·ha^−1^ in the WW condition and 7.67 m^3^·ha^−1^ in WS, representing a reduction of 77.16%; in turn, the GY expressed an estimate of 1560.43 Kg·ha^−1^ in WW and 420.03 Kg·ha^−1^ in WS, with a reduction of 73.08%, while there was a reduction of 23.24% for the EC, with averages of 21.73 mL·g^−1^ in WW and 16.68 mL·g^−1^ in WS (Figure 4).

Concerning the grain size traits, these showed a smaller magnitude of reduction when comparing the WW and WS conditions, with values of less than 10%. There was a 2.94% increase in the circularity index (CCI) and a 1.30% increase in the thickness/width ratio (TWR) (Figure 4).

### 3.2. Study of Genotypic Correlations between Agronomic Traits

**Figure 5:**
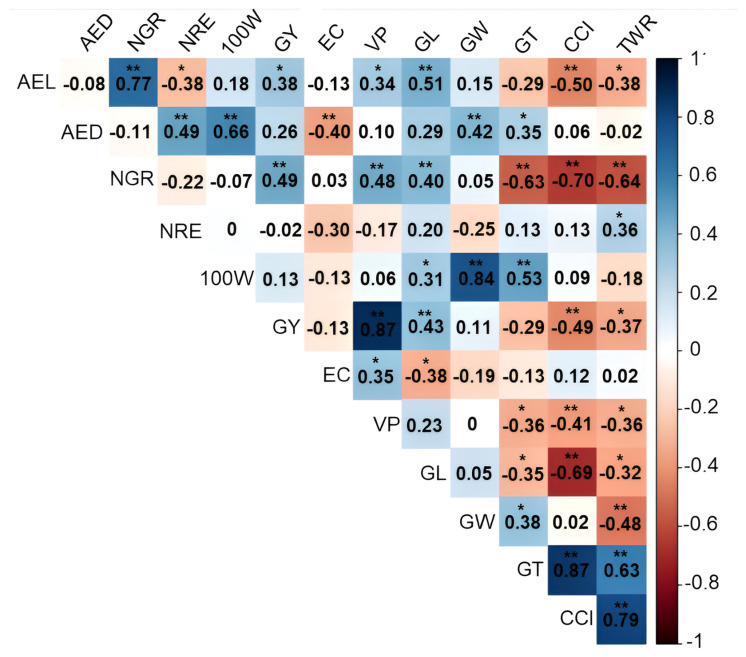
Estimates of the genotypic correlation coefficient (*rg*) represented by a heatmap between 12 agronomic variables evaluated in 43 popcorn lines under well-watered conditions (WW). * and **, significant at *p* < 0.05 and significant at *p* < 0.01, respectively, by the F test. AEL: average ear length; AED: average ear diameter; NGR: number of grains per row; NRE: number of grain rows per ear; 100 W: 100-grain weight; GY: grain yield; EC: expansion capacity; VP: volume of popcorn expanded per hectare; GL: grain length; GW: grain width; GT: grain thickness (mm); CCI: caryopsis circularity index; TWR: thickness/width ratio.

Under WW conditions, the estimates of the genotypic correlation coefficient (*rg*) with the GY character were significant and of positive magnitudes for the VP (0.87 **), NGR (0.49 **), GL (0.43 **) and AEL (0.38 *) (Figure 5). In the same WC, a negative and significant GY was observed with the CCI (−0.49 **) and TWR (−0.37 *) (Figure 5). The other ear-related traits showed a significant positive *rg* between AEL × NGR (0.77 **), AEL × VP (0.34 *), AED × NRE (0.49 **), NGR × VP (0.48 **), and EC × VP (0.35 *), and a negative *rg* between AEL × NRE (−0.38 *) and AED × EC (−0.40 **) (Figure 5).

Considering the traits related to the grain shape evaluated in the WW condition, the *rg* estimates were significant and of positive magnitudes between GL × AEL (0.51 **), GW × AED (0.42 **), GT × AED (0.35 *), GL × NGR (0.40 **), TWR × NRE (0.36 *), GL × 100 W (0.31 *), GW × 100 W (0.84 **), GT × 100 W (0.53 **), GT × GW (0.38 *), CCI × GT (0.87 **), TWR × GT (0.63 **) and TWR × CCI (0.79 **). Negative *rg* estimates occurred between CCI × AEL (−0.50 **), TWR × AEL (−0.38 **), GT × NGR (−0.63 **), CCI × NGR (−0.70 **), TWR × NGR (−0.64 **), GL × EC −0.38 *), GT × VP (−0.36 *), CCI × VP (−0. 41 **), TWR × VP (−0.36 *), GT × GL (−0.35 *), CCI × GL (−0.69 **), TWR × CG (−0.32 *) and TWR × GW (−0.48 **).

**Figure 6:**
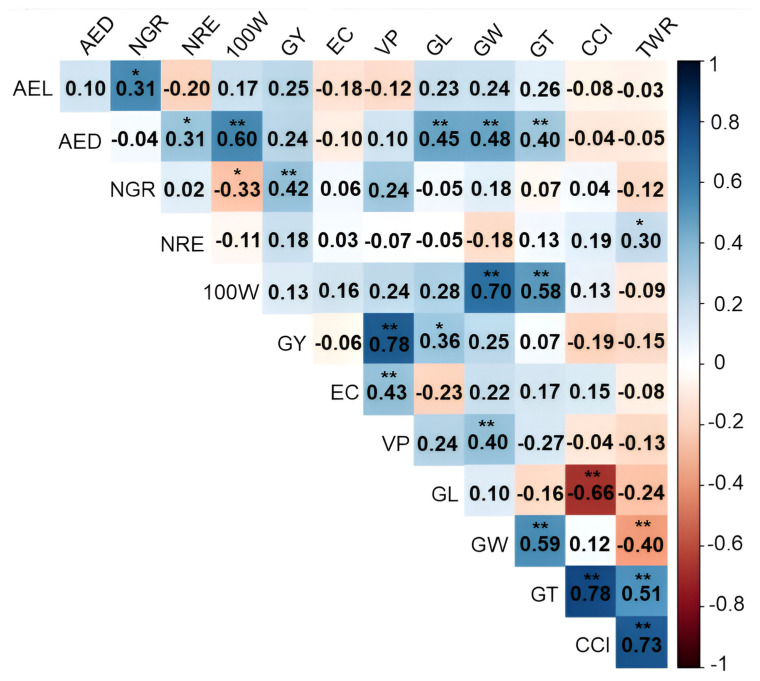
Estimates of the genotypic correlation coefficient (*rg*) represented by a heatmap between 12 agronomic variables evaluated in 43 popcorn lines under drought stress (WS). * and **, significant at *p* < 0.05 and significant at *p* < 0.01, respectively, by the F test. AEL: average ear length; AED: average ear diameter; NGR: number of grains per row; NRE: number of grain rows per ear; 100 W: 100-grain weight; GY: grain yield; EC: expansion capacity; VP: volume of popcorn expanded per hectare; GL: grain length; GW: grain width; GT: grain thickness; CCI: caryopsis circularity index; TWR: thickness/width ratio.

In WS, for the RG character, there was a positive and significant *rg* between GY × VP (0.78 **), GY × GL (0.36 *), and GY × NGR (0.42 *). The EC was positively correlated with only the VP (0.43 **) (Figure 5). For the other traits, positive and significant correlations were observed between AEL × NGR (0.31 *), AED x NRE (0.31 *), and AED × 100 W (0.60 **), and negative correlations between NGR × 100W (−0.33 *) (Figure 6).

Concerning the grain size traits, there was a significant positive correlation between AED × GL (0.45 **), AED × GW (0.48 **), AED × GT (0.40 **), 100 W × GW (0.70 **), 100 W × GT (0.58 **), NRE × TWR (0.30), VP × GW (0.40 **), GW × GT (0.59 **), GT × CCI (0.78 **), GT × TWR (0.51 **) and CCI × TWR (0.73 **), and negative correlation between GW × TWR (−0.40 **) and GL × CCI (−0.66 **) (Figure 6).

### 3.3. Leaf Indices at Different Phenological Stages, from Pre-Anthesis to Physiological Maturity

**Figure 7:**
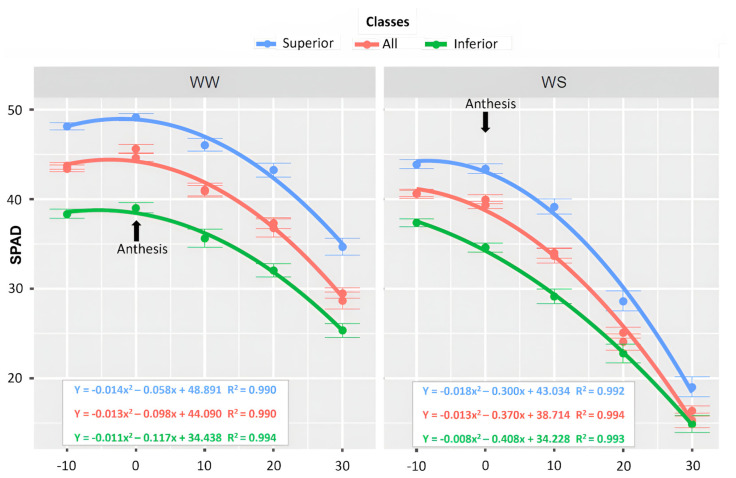
Relative chlorophyll content (SPAD), by WC, evaluated in 43 popcorn lines under WW and WS conditions, classified as superior, all, and inferior lines, conducted in 5 evaluations, from male pre-anthesis to physiological maturity (CODE 61, state 6, BBCH Scale = 0).

Concerning the relative chlorophyll content (SPAD), the superior lines (L332, L503, L625, L326, L481, L655, L391, L291, L476, and L213) had mean estimates of 48.89 and 43.03 for β0 at the initial point, considering male flowering (MF), in WW and WS, respectively (Figure 7). The maximum value of the SPAD index was 49.95 in WW and 44.27 in WS, as reached at 2 and 8 days before anthesis, respectively (−2 and −8 days AANT).

The average SPAD values of the inferior lines (L513, L502, L693, L386, L594, L382, L366, L322, L204 and L684) were β0 39.02 in WW and 34.60 in WS. The highest SPAD value in WW was 38.75, and in WS, it was 37.36, as reached at 5 and 10 days AANT, respectively (Figure 7). Evaluating the set of lines, the β0 estimates were 44.09 for WW and 38.71 for WS. The maximum SPAD value in WW was 44.27 and 40.62, respectively, for WW and WS, as obtained at 4 and 10 days AANT (Figure 7).

**Figure 8:**
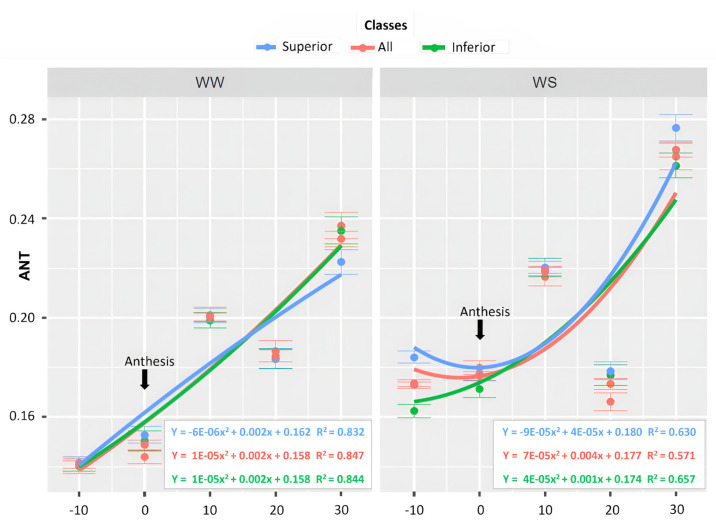
Leaf anthocyanin content (ANT), by WC, evaluated in 43 popcorn lines under contrasting water conditions, classified as superior, all, and inferior lines, conducted in 5 evaluations, from male pre-anthesis to physiological maturity (CODE 61, state 6, BBCH Scale = 0).

Concerning the ANT values, the superior lines had an estimated β0 of 0.153 in WW and 0.179 in WS. The maximum value was reached at 30 DAA (days after anthesis) for both WCs, with a magnitude of 0.230 in WW and 0.262 in WS (Figure 8). The inferior lines had an estimated β0 of 0.151 in WW and 0.169 in WS (Figure 8). The maximum value was reached at 30 DAA in both WCs. The lines showed a β0 of 0.147 in WW and 0.178 in WS. The highest magnitudes were expressed at 30 DAA in both WCs, with values of 0.233 and 0.267 in WW and WS, respectively (Figure 8).

**Figure 9:**
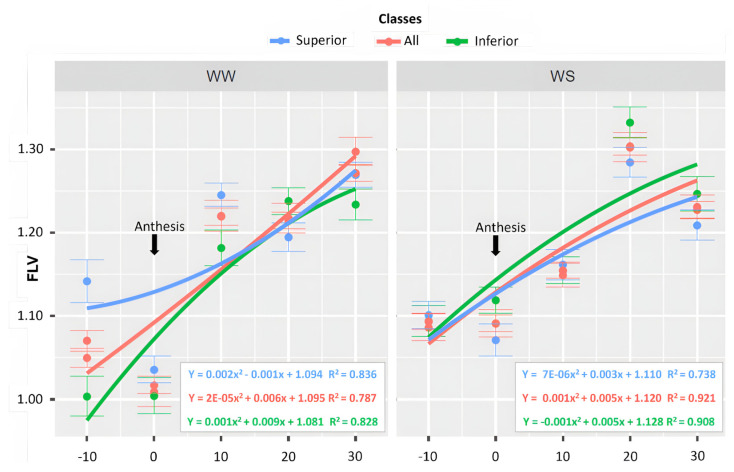
Leaf flavonoid content (FLV), by WC, evaluated in 43 popcorn lines under contrasting water conditions, classified as superior, all, and inferior lines based on 5 evaluations, from male pre-anthesis to physiological maturity (CODE 61, state 6, BBCH Scale = 0).

Concerning the FLV values, the superior lines had an estimated β0 of 1.04 in WW and 1.06 in WS. The maximum value was reached at 30 DAA for both WCs, with 1.24 in WW and 1.22 in WS (Figure 9). The inferior lines had β0 estimates of 1.00 in WW and 1.12 in WS (Figure 9). The maximum value was reached at 30 DAA in both WCs. All the lines together showed a β0 of 1.02 in WW and 1.09 in WS. The highest magnitudes were expressed at 30 DAA in both WCs, with values of 1.28 and 1.33 in WW and WS, respectively (Figure 9).

## 4. Discussion

### 4.1. Impact of Soil Water Restriction on Agronomic Traits and Grain Size

The yield components most affected by the soil water restriction were the GY and VP. The yield covariates were reduced to a lesser extent by the WS condition applied during pre-anthesis and grain filling; however, the sum of these effects drastically reduced the main traits GY and VP. In maize, the decrease in the GY under drought conditions has been attributed to the reduction in the number of grains produced per unit area [40]. In our study, the reduction in the traits related to the grain number—AEL and NGR—and grain mass—100 W—influenced the significant reduction in the GY. In a previous study, Kamphorst et al. [32] confirmed that drought stress applied between the pre-anthesis and grain-filling periods reduced the yield covariates to a lesser extent. Water deficit can shorten the grain-filling period due to reduced photosynthetic activity [41,42,43,44].

The AEL and AED traits were affected by drought stress to a lesser extent compared to the traits above, with reductions of 15.51% and 10.43%, respectively, which consequently reduced the NGR (18.07%) and NRE (11.26%). The mass of a hundred grains was also affected by the water limitation, with a reduction of 13.14%. Water deficit during fertilization can cause serious problems in grain development [45], especially in grain filling, which may have reduced the AEL, AED, NGR, and NRE in WS. Water deficit in the pre-flowering period causes the growth of the ear to be delayed relative to the growth of the shoot, resulting in a gap between the extrusion of anthers and the exposure of pollen grains to the stigma, directly affecting fertilization and, consequently, the formation of grains [46].

The grain size traits, i.e., GL, GW, and GT, showed little influence from the water deficit, as they had the smallest reductions compared to the irrigated condition. What can be seen is that the grain size was not altered under drought conditions. Thus, these traits are not a useful option for plant selection, considering that there were significant effects between G*WC, which indicates that there are different responses from the genotypes evaluated in the different WCs and, therefore, in recommending cultivars for specific environments. The grain size traits are important for determining the grain expansion capacity. Compared to the GY and VP, the EC was less affected by the soil water limitation, with proportional reductions between WCs of 23.34%. Kamphorst et al. (2020) [33], evaluating popcorn lines, recorded reductions of 29.31% and 9.66% in different harvests when comparing the WS and WW conditions; and Lima et al. (2019) [47], evaluating corn-popcorn lines and hybrids, recorded a reduction of 9.08%. The expansion process is associated with the moisture contained in the grain starch granules, which exert pressure on the pericarp when heated (≈180 °C), the rupture of which exposes the endosperm [48].

The main variables, i.e., GY, EC, and EP, were significant in the G*WC interaction, indicating different responses from the genotypes evaluated in the different WCs. Interactions of this kind impede the selection gains and the recommendation of cultivars for particular environments [49]. In these cases, correlated traits are identified for indirect selection. This is because correlated traits might have determining factors in the expression of the main traits, such as the HY, EC and PV, and still not have a significant G * WC [12]. In a study carried out by Kamphorst et al. [32], which evaluated lines and hybrids of popcorn in different crops seasons and WC, significant genotype * crop seasons, genotype * water condition, and genotype * crop seasons * water condition interactions were present.

### 4.2. Implications of Genetic Associations between Traits

Knowledge of the association between traits is significant in plant breeding. These estimates quantify the possibility of indirect gains from selection based on correlated traits. In many cases, for low heritability traits, such as the grain yield, selection is more efficient when conducted on correlated ones [50].

Under WW, the GY was significantly and positively associated with the VP, number of grains per row (NGR), average grain length (GL), and average ear length (AEL), indicating that selection based on any of these will lead to simultaneous gains in the grain yield. Under the same water deficit, on the other hand, the grain yield character (GY) was significantly and negatively associated with the caryopsis circularity index (CCI) and the thickness/width ratio (TWR), which shows that it is not possible to obtain simultaneous gains for both traits. In this sense, it can be inferred that the most productive popcorn lines in terms of the grain yield (GY) have a lower caryopsis circularity index (CCI) and grain thickness/width ratio (TWR), which likely made a contribution to the lower expansion capacity (EC). The negative correlation between the expansion capacity (EC) and grain length (GL) shows that rounder grains have a higher EC, reinforcing studies already conducted by Cabral et al. (2016) [51]. Indirect selection for the EC can be conducted by selecting genotypes with small grains; however, selecting genotypes using the caryopsis circularity index (CCI) and grain thickness/width ratio (TWR) as criteria for increasing the EC hurts the GY [52]. In the study conducted here, there was an estimated positive correlation between the expansion capacity (EC) and caryopsis circularity index (CCI) (0.12); however, it was not significant.

In the growing conditions with soil drought stress, the grain yield (GY) trait was significantly and positively associated with the VP, the NGR, and the length of the grain (GL). On the other hand, the EC was only positively correlated with the expanded popcorn volume per hectare (PV). By observing that the GY maintained a significant and positive correlation with the VP, NGR, and grain length (GL) in both water conditions evaluated, as well as between the expansion capacity (EC) and volume of VP, it follows that the latter can therefore be useful for indirect selection aimed at simultaneously increasing the GY and EC. As for the other ear and grain traits evaluated under WW, it should be noted that the gains in ear length (AEL) simultaneously resulted in a higher number of grains (NGR) and that the ears with a larger diameter (AED) tend to promote concomitant gains in the number of rows (NRE) and grain weight (100 W), reflecting in both cases simultaneous gains in the grain yield (GY).

### 4.3. Leaf Senescence Dynamics

Irrespective of the phenotypic classification (all the lines), the maximum SPAD values were reached later (4 DAAT) in WW when compared to WS (10 DAAT), and these values were also lower in drought. Obtaining the highest possible green index values and delaying senescence contribute to higher agricultural productivity since photosynthetic assimilation of carbon and the mobilization of nutrients from senescing leaves to reproductive organs are important for growth and development [53]. Regardless of the WC, the superior genotypes in terms of productivity remained their stay green for longer duration and expressed peak values later. On the other hand, the inferior genotypes reached the maximum SPAD values earlier. This is an important finding and shows an association between higher SPAD values and higher GY values since leaves with late senescence increase the period of transfer of photoassimilates to fill the grains [53].

The accessory pigments ANT and FLV are associated with the protection of the photosynthetic apparatus from damage caused by reactive oxygen species (ROS) [54,55]. The production of these pigments is associated with mitigating the harmful effects of drought in association with high solar radiation. In this context, the ANT values were consistently higher in the WS condition, demonstrating the influence of drought on the increase in these substances. Additionally, the maximum ANT values were reached at 30 DAA for both WCs, although they were higher in WS. These values indicate that leaf senescence was accelerated and influenced by the water-limited environment [56]. Similarly, the FLV values, like the ANT, were always higher in the WS conditions, demonstrating the effect of water limitation in increasing these substances. The highest FLV values were also observed at 30 DAA in both WCs.

The genotypes were categorized based on the highest GY results to discern the physiological traits inherent in the superior lines under drought conditions. The conclusion is that soil water limitation, regardless of the genotype groups, reduced the greenness index more quickly in WS. The plant reproductive stage coordinates leaf senescence but is also strongly affected by the environment [56]. The SPAD readings serve as an indicator of photochemical activity, as they exhibit a strong positive correlation with the leaf chlorophyll concentration, providing insights into the photosynthetic activity [57]. Although the ANT and FLV values increased during the water deficit, they were not effective traits for differentiating the most productive genotypes from the least productive.

## 5. Conclusions

Genetic variability and distinct behavior were detected among the popcorn lines for all the agronomic traits under the WW and WS conditions. The VP, GY, and EC traits showed the greatest reductions in WS and are indicated for genotypic discrimination under drought stress. The GY was positively correlated with the VP, NGR, and GL in both water conditions, so the latter is useful for indirect selection and is of great interest for plant breeding aimed at simultaneously increasing these traits. Water deficit influenced the decrease in leaf greenness for the stress, and as a way of adapting to the condition of water stress, the content of anthocyanins and flavonoids increased.

## Figures and Tables

**Table 1:** Precipitation and irrigation (mm) applied in the well-watered (WW) and water deficit (WS) conditions at various intervals after sowing.

Days after Sowing	Rainfall (mm)	Amount of Water (mm)			
		WW		WS	
		Irrigation Applied (mm)	Total	Irrigation Applied (mm)	Total
1	0.00	2.65	2.65	2.53	2.53
7	2.00	4.53	6.53	4.41	6.41
14	6.20	27.07	33.27	20.24	26.44
21	24.60	3.70	28.30	3.55	28.15
28	12.60	3.88	16.48	1.01	13.61
35	30.00	7.01	37.01	4.33	34.33
42	0.60	8.06	8.66	10.82	11.42
49 (CODE 61, state 6, BBCH Scale)	0.60	5.38	5.98	5.43	6.03
56	5.40	4.68	10.08	0.00	5.40
63	1.60	13.71	15.31	0.00	1.60
70	2.20	15.00	17.20	0.00	2.20
77	8.20	5.48	13.68	0.00	8.20
84	1.20	7.29	8.49	0.00	1.20
91	0.40	7.66	8.06	0.00	0.20
98	0.80	15.06	15.86	0.00	0.80
105	20.40	2.42	22.82	0.00	20.40
112	0.20	4.26	4.46	0.00	0.20
119	0.00	0.00	0.00	0.00	0.00
Total	117.00	137.84	254.84	52.31	169.31

mm: millimeters.

**Table 2:** Summary of the individual and joint analyses of variance, and estimates of the means, standard deviations, and values of the coefficients of experimental variation, for 12 agronomic traits evaluated in 43 corn popcorn lines grown under well-watered (WW) and water deficit (WS) conditions.

Traits	WC	MS	Mean + Standard Deviations	CV_e_ (%)	Joint Analyses		
		Genotype (G)			G	WC	G * WC
		(DF = 42)					
AEL	WW	7.09 **	11.09 ± 1.25	11.27	**	**	**
	WS	5.38 **	9.37 ± 1.39	14.83			
AED	WW	14.71 **	27.90 ± 1.54	5.52	**	**	**
	WS	28.37 **	24.99 ± 1.71	6.84			
NGR	WW	42.96 **	22.86 ± 3.01	13.17	**	**	**
	WS	25.92 **	18.73 ± 3.44	18.37			
NRE	WW	5.30 **	13.14 ± 1.22	9.28	**	**	**
	WS	7.50 **	11.66 ± 1.22	10.46			
100 W	WW	8.06 **	10.73 ± 1.29	11.67	**	**	*
	WS	8.79 **	9.32 ± 1.43	16.51			
GY	WW	910,769.10 **	1560.43 ± 266.23	16.74	**	**	**
	WS	150,730.26 **	420.03 ± 102.59	26.46			
EC	WW	64.50 **	21.73 ± 1.50	6.90	**	**	**
	WS	69.87 **	16.68 ± 1.64	9.83			
VP	WW	541.98 **	33.58 ± 6.37	18.97	**	**	**
	WS	78.03 **	7.67 ± 4.76	62.06			
GL	WW	1.28 **	7.19 ± 0.52	7.23	**	**	**
	WS	1.01 **	6.80 ± 0.33	4.85			
GW	WW	0.72 **	5.73 ± 0.31	5.41	**	**	**
	WS	0.59 **	5.49 ± 0.21	3.82			
GT	WW	0.59 **	4.34 ± 0.26	5.75	**	ns	**
	WS	0.49 **	4.29 ± 0.26	5.99			
CCI	WW	0.005 **	0.34 ± 0.03	8.82	**	*	**
	WS	0.004 **	0.35 ± 0.03	8.57			
TWR	WW	0.020 **	0.77 ± 0.06	7.79	**	*	**
	WS	0.014 **	0.79 ± 0.05	6.33			

**, *, ns, significant at 1%, 5%, and not significant, respectively, by the F test. MS: mean square; DF: degrees of freedom; SD: standard deviation; CV_e_: coefficient of experimental variation (%); G: genotype; WC: water condition; G * WC: genotype * water condition interaction; AEL: average ear length (cm); AED: average ear diameter (mm); NGR: number of grains per row (pcs); NRE: number of grain rows per ear (pcs); 100 W: 100-grain weight (g); GY: grain yield (Kg·ha^−1^); EC: expansion capacity (g·mL^−1^); VP: volume of popcorn expanded per hectare (m^3^·ha^1^); GL: grain length (mm); GW: grain width (mm); GT: grain thickness (mm); CCI: caryopsis circularity index; TWR: thickness/width ratio.

## Data Availability

The raw data supporting the conclusions of this article will be made available by the authors on request.

## Supplementary Materials

The following supporting information can be downloaded at https://www.mdpi.com/article/10.3390/life14060743/s1, Table S1: Description of the popcorn lines and information on the generation, country of origin, provenance, and climate adaptation.
