# Supplementary material for: Unveiling Drought-Resilient Latin American Popcorn Lines through Agronomic and Physiological Evaluation
Source: Life (Basel). 2024 Jun 11;14(6):743. doi: 10.3390/life14060743 (PMC11204607; doi:10.3390/life14060743)
Supplement: Supplementary file 1 [file life-14-00743-s001.zip › life-3012609-supplementary.pdf]

Supplementary Table S1. Description of the popcorn lines and information on generation, country of origin, provenance, and climate adaptation.

lines	Genealogy	Self-fertilization generation	Country of origin	Provenance	Climate adaptation
**L203**	IAC 125	S_7	Brasil	IAC	Tropical
**L204**	IAC 125	S_7	Brasil	IAC	Tropical
**L213**	IAC 125	S_7	Brasil	IAC	Tropical
**L217**	IAC 125	S_7	Brasil	IAC	Tropical
**L219**	IAC 125	S_7	Brasil	IAC	Tropical
**L220**	IAC 125	S_7	Brasil	IAC	Tropical
**L221**	IAC 125	S_7	Brasil	IAC	Tropical
**L222**	IAC 125	S_7	Brasil	IAC	Tropical
**L263**	PARA 172	S_7	Paraguai	CIMMYT	Temperate
**L273**	PARA 172	S_7	Paraguai	CIMMYT	Temperate
**L291**	URUG 298	S_7	Uruguai	CIMMYT	Temperate
**L292**	URUG 298	S_7	Uruguai	CIMMYT	Temperate
**L321**	UFV M-2 Barão de viçosa	S_7	Brasil	UFV	Tropical
**L322**	UFV M-2 Barão de viçosa	S_7	Brasil	UFV	Tropical
**L326**	UFV M-2 Barão de viçosa	S_7	Brasil	UFV	Tropical
**L328**	UFV M-2 Barão de viçosa	S_7	Brasil	UFV	Tropical
**L332**	UFV M-2 Barão de viçosa	S_7	Brasil	UFV	Tropical
**L366**	PR 023	S_7	Brasil	UEM	Tropical
**L381**	SAM	S_7	EUA	EUA	Temperate
**L382**	PR 023	S_7	Brasil	UEM	Temperate
**L383**	SAM	S_7	EUA	EUA	Temperate
**L386**	SAM	S_7	EUA	EUA	Temperate
**L391**	SAM	S_7	EUA	EUA	Temperate
**L472**	SE 013	S_7	Brasil	UEM	Tropical
**L476**	SE 013	S_7	Brasil	UEM	Tropical
**L480**	SE 013	S_7	Brasil	UEM	Tropical
**L481**	SE 013	S_7	Brasil	UEM	Tropical
**L501**	PA 170 Roxo	S_7	Paraguai	CIMMYT	Temperate
**L502**	PA 170 Roxo	S_7	Paraguai	CIMMYT	Temperate

**L503**	PA 170 Roxo	S_7	Paraguai	CIMMYT	Temperate
**L507**	PA 170 Roxo	S_7	Paraguai	CIMMYT	Temperate
**L509**	PA 170 Roxo	S_7	Paraguai	CIMMYT	Temperate
**L510**	PA 170 Roxo	S_7	Paraguai	CIMMYT	Temperate
**L513**	PA 170 Roxo	S_7	Paraguai	CIMMYT	Temperate
**L594**	RS 20	S_7	Brasil	IPAGRO/ AGROESTE	Temperate
**L61**	BRS Ângela	S_7	Brasil	EMBRAPA	Tropical
**L625**	PA 091	S_7	Brasil	UEM	Tropical
**L652**	ARZM 13 050	S_7	Argentina	CIMMYT	Temperate
**L655**	ARZM 13 050	S_7	Argentina	CIMMYT	Temperate
**L684**	UENF 14	S_7	Brasil	UENF	Tropical
**L688**	UENF 14	S_7	Brasil	UENF	Tropical
**L689**	Viçosa-Viçosa	S_7	Brasil	UFV	Tropical
**L691**	UENF 14	S_7	Brasil	UENF	Tropical
**L693**	UENF 14	S_7	Brasil	UENF	Tropical

MF: Male Flowering (days); USA: United States of America; UFV: Federal University of Viçosa; EMBRAPA: Brazilian Agricultural Research Corporation; IAC: Agronomic Institute of Campinas; CIMMYT: International Maize and Wheat Improvement Center; UEM: State University of Maringá; IPAGRO: Agronomic Research Institute; and UENF: State University of Norte Fluminense Darcy Ribeiro.
